# Contemporary techniques of da Vinci SP radical prostatectomy: multicentric collaboration and expert opinion

**DOI:** 10.1590/S1677-5538.IBJU.2022.99.16

**Published:** 2022-03-30

**Authors:** Marcio Covas Moschovas, Isabella Brady, Jonathan Noel, Mahmoud Abou Zeinab, Aaron Kaviani, Jihad Kaouk, Simone Crivellaro, Jean Joseph, Alexandre Mottrie, Vipul Patel

**Affiliations:** 1 AdventHealth Global Robotics Institute Celebration USA AdventHealth Global Robotics Institute (GRI), Celebration, USA;; 2 University of Central Florida Orlando USA University of Central Florida (UCF), Orlando, USA;; 3 Glickman Urological & Kidney Institute Cleveland Clinic Cleveland USA Glickman Urological & Kidney Institute, Cleveland Clinic, Cleveland, USA;; 4 University of Illinois Chicago USA University of Illinois, Chicago, USA;; 5 University of Rochester New York USA University of Rochester, New York, USA;; 6 OLV Hospital ORSI Academy Aalst Belgium OLV Hospital/ ORSI Academy, Aalst, Belgium

**Keywords:** Robotic Surgical Procedures, Prostatectomy, methods [Subheading]

## Abstract

**Background:**

The da Vinci SP robot consists of an innovative single port trocar that houses a flexible camera and three biarticulated arms, which minimizes the number of incisions to assess the surgical site, allowing a less invasive procedure. However, due to its recent release in the market, the current literature reporting SP-RARP is still restricted to a few centers. In this scenario, after performing a literature search with all available techniques of SP-RARP, our objective is to report a multicentric opinion of referral centers on different techniques to approach SP-RARP.

**Results:**

The SP literature is provided by only a few centers due to the limited number of this new console in the market. Five different approaches are available: transperitoneal, extraperitoneal, Retzius-Sparing, transperineal and transvesical. None of the current studies describe long-term functional or oncological outcomes. However, all approaches had satisfactory operative performance with minimum complication rates.

**Conclusions:**

Several techniques of SP-RARP have been reported in the literature. We performed a multicentric collaboration describing and illustrating the most challenging steps of this surgery. We believe that the details provided in this article are useful teaching material for new centers willing to adopt the SP technology.

## INTRODUCTION

The da Vinci Robot was first introduced into urologic surgery in the United States in 1999 after FDA approval ( 1 ). Since then, robotic-assisted radical prostatectomy (RARP) using the multi-port system has developed into the gold standard for surgical management of prostate cancer in the USA. In this scenario, during several da Vinci generations, urologists and robotic surgeons continue to develop minimally invasive techniques to reduce morbidity and maximize outcomes. As a result, surgical times, intraoperative performances, complication rates, and postoperative outcomes have improved drastically.

After numerous multiport consoles, the first da Vinci single port (SP) clinical investigation system in urology was reported in December 2014 by Kaouk et al. ( 2 ), although the Food and Drug Administration (FDA) approved selling the SP system only a few years later, in November 2018 ( 3 ). The new SP robot incorporates a single port that houses a flexible camera and three biarticulated arms, which minimizes the number of incisions required to assess the surgical site, allowing a less invasive procedure ( 4 ). However, due to its recent release in the market, the current literature reporting SP RARP is still restricted to a few centers. Therefore, the aim of this study is to report the experience and opinion of SP referral centers regarding crucial aspects of this platform on radical prostatectomies.

## PATIENTS AND METHODS

On July 25th, 2021, during the Society of Robotic Surgery (SRS) annual meeting, referral centers on SP surgery discussed crucial aspects of the SP approach to radical prostatectomy. Each center shared their experience and challenges from the da Vinci SP implementation until the operative routine after achieving the learning curve. We have described in detail the critical aspects of this consensus on each surgical approach of this article.

## RESULTS

### SP system implementation

#### Training for SP surgery (animal and cadaver), simulator and certification

The training for SP surgery relates to the initial background of the surgeons. A faster learning curve is expected for surgeons with previous robotic experience, but such a learning curve continues to exist. For non-robotic surgeons, the learning curve is usually steeper. SP system training is a must for all surgeons before implementing SP applications. It starts with a didactic dry lab course on how to use the robot in terms of joysticks, pedals, and the functionality of controls.

The next step is an optional wet lab training, if possible, followed by taking advantage of several courses with SP experts to learn the landscape and expected outcomes. Tips and tricks from surgeons already using the SP platform are also useful. The next stage is case observation of SP procedures, suggesting around 5 cases, followed by performing at least 2-3 select cases in a proctored fashion. The final stage of SP training and implementation is performing SP surgeries with an experienced SP robotic surgeon being available if needed. Certificates of proficiency should be issued upon training by program directors of the corresponding institutes.

## Selection criteria for SP-RARP

The patients should be always informed about the learning curve experience. Cases should be selected in a fashion that is less complex before progressing into the more complex pathologies. Body habitus should be selected to be favorable, and a start with an easier procedure such as pelvic surgeries (simple or radical prostatectomy) is recommended. Favorable pathologies such as low or intermediate-risk prostate cancer should be first selected before proceeding to higher-risk patients. Finally, establishing a local database to track outcomes helps in optimizing future patient selection criteria and technical adjustments.

## Floating trocar technique and considerations

**Figure 1:**
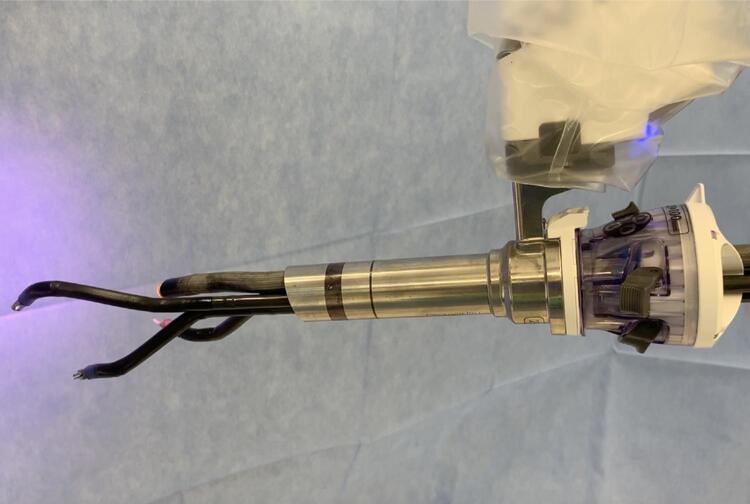
SP trocar and instruments attached to the robotic arm.

The single port platform has been originally designed to be used mostly in the peritoneal cavity. The single metallic trocar was supposed to be inserted through the fascia all the way into the peritoneum ( Figure-1 ). This approach though poses multiple issues. Having the trocar completely inserted doesn’t allow to perform “pure” single port surgery given the need of an extra trocar for suction. Additionally, the single port instruments require at least 10 cm of distance from the tip of the trocar to articulate. Therefore, the trocar inside the cavity makes it virtually impossible to efficiently work in shallow spaces such as the retro or extra peritoneal. A specific way of docking called “floating dock” allow to overcome this issue.

**Figure 2:**
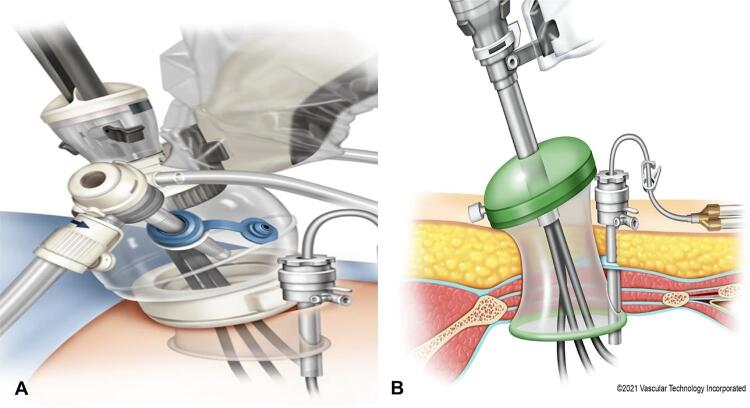
A) SP Access Kit (Intuitive). B) Mini Gel Point (Applied Medical) with an assistant trocar placed laterally.

Essentially the trocar is docked outside of the cavity and it “floats” exterior to the abdomen giving the chance to perform “pure” single port surgery while working in small, shallow spaces. To efficiently perform the “floating dock”, two different devices can be used: the Mini GelPOINT (Applied Medical) or the SP access port (Intuitive) as illustrated by Figure-2A and 2B .

In both cases the trocar is docked at least 10 cm away from the skin level, therefore allowing the instruments to enter the cavity and articulate almost immediately. To make the floating dock technique more efficient and avoid instruments, both the camera and flexible suction pass through the same incision as a “sidecar” trocar as depicted in Figures 1 and 2 . This trocar can be a 5 or a 12 mm and is essentially placed through the same skin incision, different fascial incision and eventually through the retractor of the Mini Gel Point or the SP Access Port under direct digital control.

## Single Port Transperitoneal considerations

**Figure 3:**
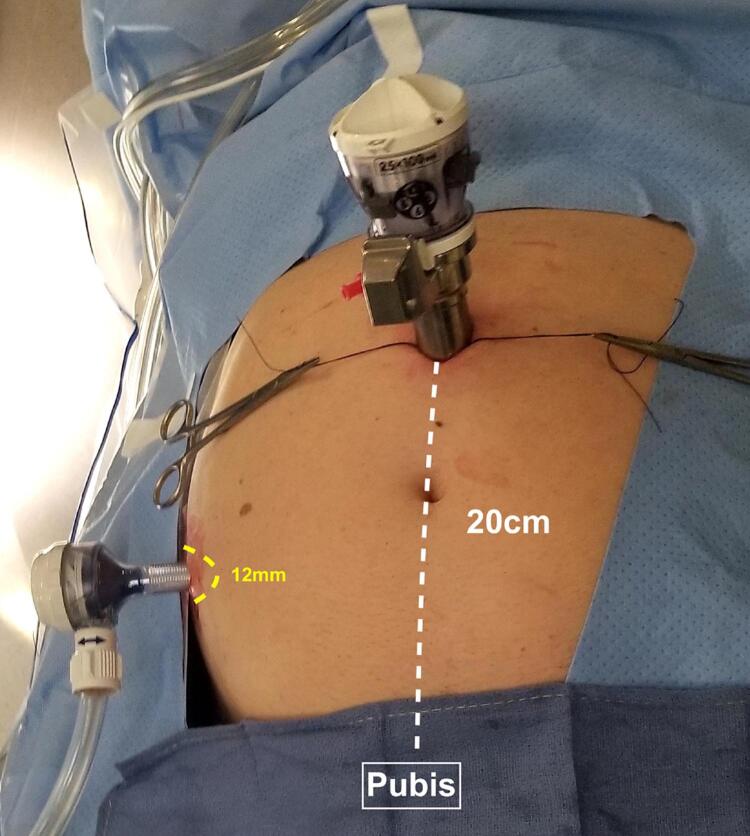
SP+ one transperitoneal trocar placement.

Transperitoneal access is one of the options to approach SP-RARP. With this technique, we usually place the robotic trocar on the midline 15 to 20cm from the pubic bone using Hasson’s technique ( Figure-3 ) ( 5 , 6 ). However, several techniques of transperitoneal SP-RARP have been described using infraumbilical incision and “floating trocar” ( 7 , 8 ).

The transperitoneal approach allows a full access to the abdominal cavity without space restrictions or limitations to perform an extended lymphadenectomy. It allows accessing multiple quadrants while minimizing technique modifications from the multiport RARP technique, being the easiest and recommended transition from the multiport to the SP robot. However, transperitoneal surgery can face challenges in patients with several previous procedures and complex bowel adhesions.

## SP Transperitoneal Port placement

In general, two types of port placement have been described in transperitoneal SP-RARP. One type is the “pure SP” which is usually placed with the GelPOINT (Applied Medical) or the Intuitive access kit. The second type is the “SP plus one” which typically does not require auxiliary devices and allows easier transition from multiport to the pure SP due to minimal modifications in surgical technique and minimal increase in operative time. It is also associated with reduced intraoperative disposable costs ( 9 ).

## Transperitoneal SP-RARP Technique

The SP robot imposes some technical modifications and a new learning curve to approach new camera settings and instrument modifications. However, the surgery follows the same concept and steps described in previous series of multiport RARP ( 10 - 15 ).

1. Patient positioning and trocar placement (Single port plus one);
2. Bladder dropping and Retzius space access;
3. Anterior bladder neck dissection;
4. Posterior bladder neck dissection and seminal vesicles approach;
5. Nerve sparing (posterior access and lateral dissection);
6. Prostatic pedicles control with Hem-o-lock clips;
7. Minimal apical dissection;
8. DVC control with running suture and urethra division;
9. Posterior reconstruction and anastomosis;
10. Lymphadenectomy.

## Single Port Extraperitoneal considerations

Extraperitoneal robot assisted radical prostatectomy aims to duplicate the previously known “gold standard” open radical retropubic prostatectomy. In the latter, a midline incision provides direct access to the target organ upon entry into the space of Retzius. While Multiport robotic surgery continues to replace open radical prostatectomy at most centers, Single Port robotic prostatectomy promises to truly replicate the open approach when performed extraperitoneally using a small (<3cm midline) incision, with the added accuracy of the robotic technology, and further limitation of the surgical invasiveness.

We have developed a reproducible technique to develop the extraperitoneal space for multiport, which we have adapted to the single port robot. Briefly, we use a 3 cm midline incision, about 5 cm from the umbilicus and exposing the linea alba which is entered. Once the perivesical fat is identified, a balloon dilator is inserted to the level of the pubic symphysis. With a camera placed in the balloon dilator, the space is created under direct vision. Important landmarks include the pubic symphysis caudally, the epigastric vessels anteriorly. No additional dilation is necessary once the epigastric vessels are visualized. When using a “Plus One” technique, the additional trocar can be placed directly into the balloon dilator. Alternatively, the surgeon can place a finger through the midline incision, over which the additional trocar is guided. In addition, the “dreaded peritoneotomy” which can result from the balloon dilation is not encountered. A peritoneotomy is common in cases of prior mesh inguinal hernia repair, appendectomy, or other interventions causing scarring of the peritoneum which can lead to tearing when stretched by the balloon.

A GelPOINT mini (Applied Medical) or, or an SP Access kit is used to create the pneumoretroperitoneum, allowing visualization of the working space. We prefer using the SP access kit due to several advantages. The instruments can be visualized as they pass through the wound retractor, given the transparent balloon extending the insufflated working space. The docking port is extended with the port of entry naturally floated over the inflated access kit balloon.

Due to technological limitations and a resulting long learning curve, most surgical teams embarking on robotic prostatectomy chose the transperitoneal route, where the anatomy is more easily recognizable. The transperitoneal route, except in a “Retzius Sparing” approach requires a “bladder take down step” to access the prostate. The ease of creating a smaller working space for the multiport access will lead to more surgeons choosing the extraperitoneal route when using the SP. Additional instrumentations, and refinement of the single port robot will undoubtedly continue and lessen the invasiveness of surgical intervention which we all strive for.

## Single Port Retzius-sparing

**Figure 4:**
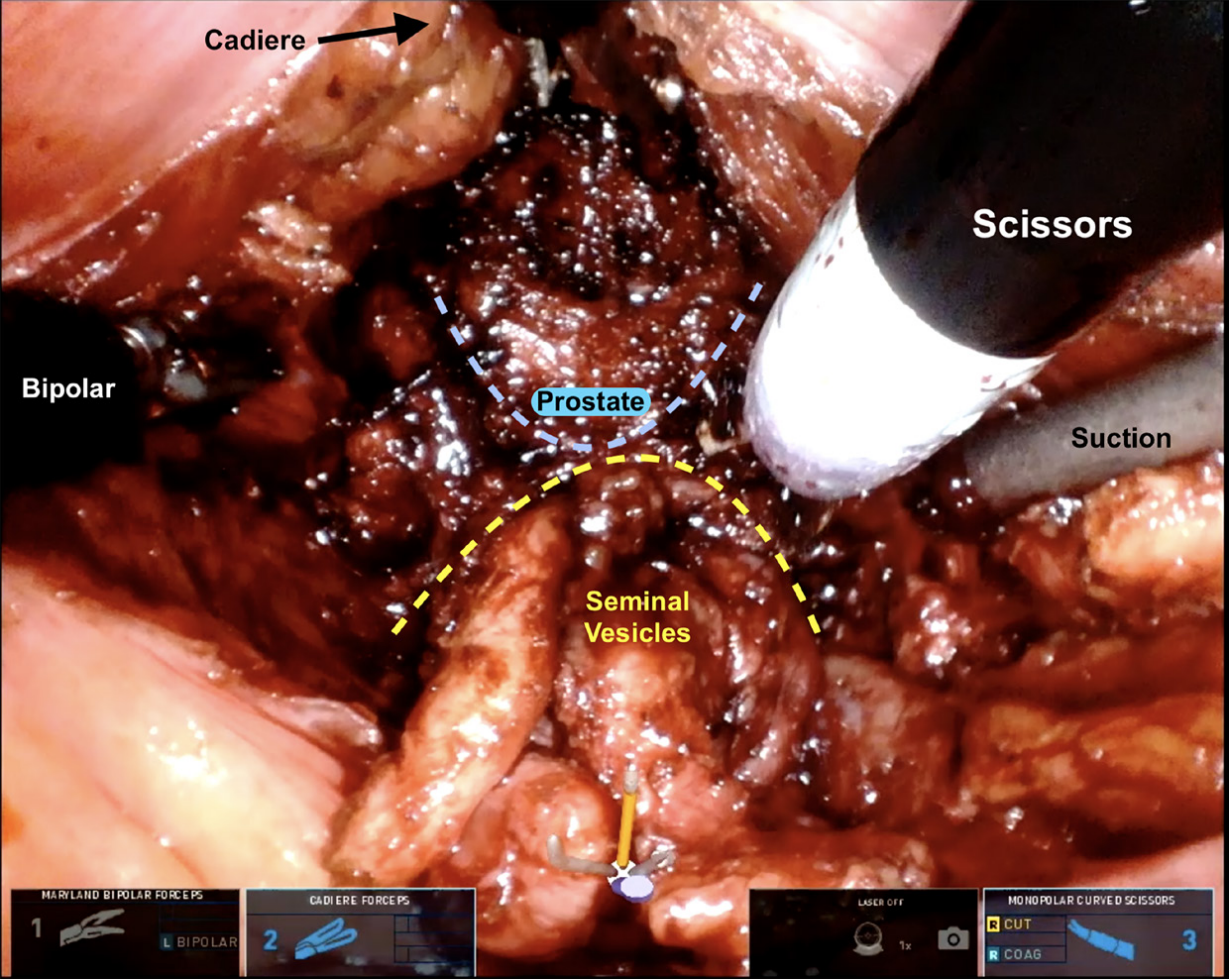
SP Retzius-sparing: anterior bladder neck approach (Cadiere at 12 holding the bladder

Retzius-sparing robotic radical prostatectomy has been originally described by Galfano et al. ( 16 ), to remove the prostate while preserving the periprostatic organs and structures including the bladder, the deep venous complex, the endopelvic fascia, the puboprostatic ligaments, and all the other structures in the anterior compartment ( 17 ). The technique has been associated with overall improved urinary continence rates compared to anterior approaches and immediate continence after catheter removal described up to 92% of patients ( 18 , 19 ). The single port (SP) robotic platform has been designed to work in small, tunnel like spaces. Given its unique flexible camera, the proximal articulation, and the single-entry point of all the instruments, SP provides an exceptional possibility of working efficiently in “hard to reach” anatomic locations. Because of these peculiarities it might be uniquely suited to the anatomy of the recto-vesical pouch and Retzius sparing approach ( Figure-4 ). Beside the already reported advantages in terms of decreased pain, shorter length of stay, and improved cosmesis, the SP might be able to facilitate the Retzius sparing technique and therefore adding advantages in terms of faster urinary continence recovery. A multicentric report of our initial experience with the single-port platform for a Retzius-sparing approach to radical prostatectomy is under review and proves safety, feasibility, and comparable oncological and functional outcome with the reported multiport results. Further studies are on the way to compare intra and perioperative outcomes of SP versus multiport Retzius sparing prostatectomy.

## Single-port Transvesical Approach

The single-port transvesical RARP approach was developed after we established our experience with SP transvesical robot-assisted simple prostatectomy and SP extraperitoneal RARP. Patients with localized prostate cancer and a history of extensive abdominal surgeries such as ( 20 ) colectomy with a colostomy or J-pouch creation, as well as those with NCCN (National Comprehensive Cancer Network) low to intermediate-risk disease were selected for this approach ( 21 ).

Patients are placed in a supine position; a 3.5 cm suprapubic midline incision is made two fingerbreadths cephalad to the symphysis pubis. After incising the fascia, splitting the rectus muscle, identifying the bladder, and placing 3-0 Vicryl stay sutures bilaterally, a 2 cm cystotomy is made. The white internal ring of the SP access port kit wound retractor (Intuitive Surgical, California, United States) is inserted into the bladder, the sliding ring is slid down to the skin level, and the rolling ring is rolled over the sleeve to reach the sliding ring and nest into it.

**Figure 5:**
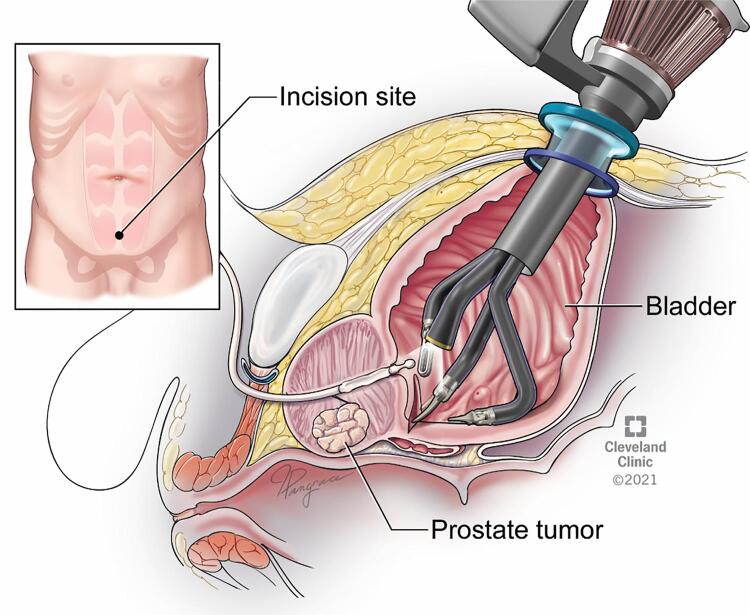
Single-port transvesical robot-assisted radical prostatectomy. The patient is kept in a supine position. The camera and instruments are introduced directly into the urinary bladder.

The 25 mm short entry guide is inserted into the access port. An 8 mm AirSeal port (Conmed Linvatec, Largo, Florida, USA) is inserted into the chamber seal. A remotely operated suction irrigation (ROSI) device (Vascular Technology, Nashua, NH, USA) is inserted. The bladder is insufflated to 12 mmHg pressure and the robot is docked ( Figure-1 ). The Instruments are illustrated by Figure-5 .

The posterior bladder neck is incised from 5 to 7 o’clock position while keeping a safe distance from the ureter orifices. The tips of seminal vesicles are clipped, and after lifting the seminal vesicles and vas deferens, Denonvilliers fascia is incised, and the posterior plane is developed between the prostate and rectum. The anterior wall of the bladder neck is then incised, and dissection is continued anteriorly.

In sequence, the endopelvic fascia is opened, puboprostatic ligaments are transected and the dorsal vein is ligated. Lateral prostatic fascia is opened bilaterally, and the vascular pedicles are identified. Pedicles are ligated then using Weck clips. The dorsal vein complex is transected, and any bleeding vein is oversewn. The urethra is divided just distal to the apex of the prostate. Once prostate dissection is completed, it is placed in the bladder. Limited lymph node dissection is performed for patients with >7% risk of lymph node involvement calculated using Briganti nomogram ( 22 ).

Next, the bladder insufflation pressure is decreased from 5 to 8 mmHg, and an 8 inch, dyed 3-0 V-Loc suture (Covidien, Mansfield, MA) is used for posterior reconstruction. The vesicourethral anastomosis is continued with the same suture in a running fashion. Another undyed suture is used for the contralateral side and both sutures are tied together at 12 o’clock. A new 20-Fr Foley catheter is inserted. The robot is undocked, and the bladder is closed in 2 layers. Fascia is closed with 0 Vicryl sutures.

SP transvesical radical prostatectomy is an alternative novel approach for patients with a hostile abdomen and those with low or intermediate-risk disease. Patients can be discharged home on the same day and benefit from the minimal opioid requirement, shorter catheter duration, and earlier return of continence without compromising intraoperative and oncological outcomes.

## Single-Port Transperineal Approach

Due to its narrow profile, the purpose-built SP platform allows for procedures in narrow working spaces. SP-RARP using the perineal approach was developed and offered to patients who are not otherwise candidates for the traditional retropubic robotic approaches ( 23 ). Patients with extensive prior abdominal or pelvic surgeries such as total proctocolectomy and J-pouch, previous pelvic radiotherapy, or kidney transplants are offered the perineal approach to avoid working in a hostile abdomen.

**Figure 6:**
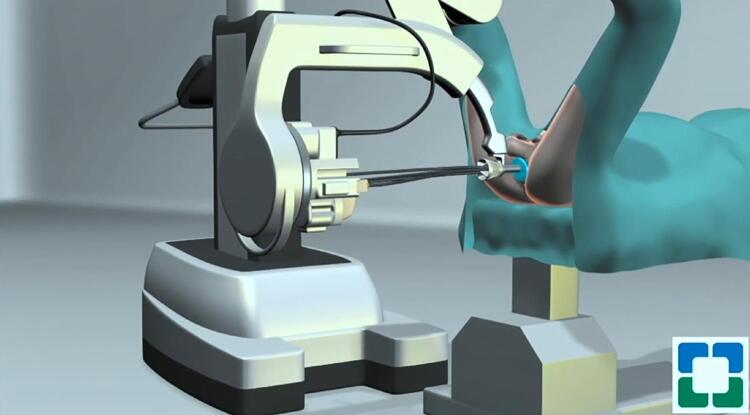
Single-port transperineal robot-assisted radical prostatectomy. An Illustration of the SP robot docked to the perineum, while the patient is in a high lithotomy position.

Patients are positioned in a high lithotomy position. A 3cm semilunar perineal incision is made. After developing the subcutaneous space between the rectourethralis and levator ani muscles, the SP robot is docked using the GelPOINT (Applied Medical, California, USA) ( Figure-6 ) ( 24 ). After exposing the levator ani muscle fibers, the Denovilliers are identified and incised, developing the posterior plane towards the base of the prostate. Next, lateral dissection is performed, and the vascular pedicle and neurovascular bundles are exposed and clipped using the robotic clip applier. The seminal vesicles and vas deferens are then identified and dissected. Using the tip of the seminal vesicle as a retractor. The release of the neurovascular bundle continues apically using sharp dissection, avoiding the use of electrocautery. Next, the membranous urethra is sharply divided starting from the posterior urethral plate. Care is required during this step since it is a common site for positive surgical margins. The dissection continues anterolaterally until the bladder is reached. Using the Foley balloon as a guide, the anterior bladder neck is opened and the dissection proceeds in a circumferential fashion and the prostate s freed from the last attachment. The robot is undocked to remove the specimen. In the perineal approach, lymph node dissection is performed in a caudal-to-cranial direction, as opposed to the conventional lymph node dissection. The obturator nerve and vein are identified first, and the dissection proceeds anterolateral to expose and dissect the obturator and external iliac lymph nodes. The vesicourethral anastomosis is completed using two 4-0 barbed running sutures in a water-tight fashion. Being the anastomosis above the camera in the perineal approach, it begins anteriorly and proceeds posteriorly. A pelvic drain is not placed in most of our cases.

The Perineal approach is considered an alternative but challenging therapeutic choice for patients with limited surgical options (frozen pelvis). It is associated with a shorter hospital stay, higher early continence rates due to the Retzius sparing approach, faster sexual recovery, and equivalent oncologic outcomes compared to the standard RARP ( 23 , 25 ).

## DISCUSSION

Before any comparisons with the multiport platform or between the SP centers, it is crucial to note that the current data has multiple confounding factors. All articles to date are based on retrospective data evaluation and their inherent risks of bias. In addition, we still don’t have a standardized technique because all centers perform this surgery with different ways of trocar placement, several types of abdominal accesses, diverging surgical techniques, and distinctive postoperative routines. Furthermore, some centers adopted selection criteria for all patients, while other surgeons only selected patients in the first cases during the learning curve. Therefore, we have explained all crucial factors and technical details that are consensus among referral centers on SP surgery.

Every surgical innovation imposes challenges on surgeons, fellows, residents, and nurses. The new SP robot, with its unique structure and features, as well as the different surgical implementations, necessitates a new learning curve for its users. A previous study performed on SP extraperitoneal RARP learning curve, identified four different learning phases until the mastery level. Low PSM rate, postoperative complications, and BCR can take time to be achieved even for experienced robotic surgeons ( 26 ).

Different factors are associated with the SP learning curve ( 15 ). We believe that the best initial approach is to select cases with favorable BMI, prostate size, and tumor staging to reduce operative time and minimize positive surgical margins (PSM). Then, after achieving proficiency, despite the surgical technique, the surgeon should choose the best approach that fits the patient’s needs in terms of cancer control and potential anatomical limitations to access the surgical field.

Finally, as previously explained, the SP robot is restricted to a few centers due to the short period in the market and some challenges posed by this platform in terms of modifications in the surgical approach and the new learning curve required. Therefore, we believe that sharing the experience of several referral centers is crucial to provide information to surgeons willing to perform a safe transition from the multiport to the SP approach. Therefore, we provided innovative teaching material and illustrations with essential aspects from the implementation until the surgical technique variation.

## CONCLUSIONS

Several techniques of SP-RARP are available in the literature, despite the short period of this robot in the market. We performed a multicentric collaboration describing and illustrating the most challenging steps of this surgery, from the technical implementation to the learning curve in different approaches. We believe that the details provided in this article are useful teaching material for new centers willing to adopt the SP technology. The available data describes feasible and safe procedures with acceptable perioperative and short-term outcomes. However, the SP literature is based on retrospective data, which carries the inherent risks of bias. In this scenario, better-designed studies with long-term follow-up are still awaited.
